# False Impressions

**Published:** 1889-01-05

**Authors:** Caroline Fothergill

**Affiliations:** Author of "An Enthusiast," "A Voice in the Wilderness," etc.


### FALSE IMPRESSIONS.

By Caroline Fothergill, Author of "An Enthusiast," "A Voice in the Wilderness," etc.

*(Continued from p. 189.)*

### Chapter XIII.—Mrs. Lester Rebels.

It was late when George got home after leaving Beatrice; he had only just time to dress before dinner, and Mrs. Lester had begun to fidget when he came in. His jubilant frame of mind had lasted all through his walk home, and it dwelt with him now. He was cheerful beyond his wont; conversed gaily with Edith, and even sustained a tolerably animated conversation with his sister.

He talked partly to keep himself from thinking; his mind dwelt persistently on one subject alone, and if he had been silent he must have become absorbed in thoughts of the scene he had witnessed such a short time ago; so he talked to keep himself free.

When the ladies left him, and the need for keeping up appearances was over, his face relaxed; he leaned back in his arm-chair and went over everything once again in his mind. He sat longer than usual over his wine, not emptying his glass even, but thinking thoughts which made him alternately smile and frown. At last he drank out his glass at a draught, rose, and sauntered across the hall, stopping for a moment at the open front door, and then passing on to join Mrs. Lester and Edith in the drawing-room.

He came into a room almost as silent as the one he had just left, where he had been sitting alone. Mrs. Lester was lying on a couch, enjoying her after-dinner nap, the book over which she had fallen asleep open on her lap. Edith sat somewhat apart in a window, and although she did not move when George came into the room, she was intensely conscious that he had come in, and her heart began to beat very fast as, without the slightest pause or hesitation, he proceeded to make his way to where she sat.

She was on a little couch, and he came up to her saying—

"Can you not make room for me on your seat?"

She moved without speaking. She felt very happy this evening, with a vague dreamy happiness, which yet left nothing to be desired. Harry Fairfax had just been in, about some parish matter in which they were both interested. It was during his visit that Mrs. Lester had fallen asleep, and he had taken advantage of their having been practically alone to press his attentions on Edith more than he had ever done before. She had at last become conscious that he was paying her attentions, and they were not disagreeable to her, nor did she behave as if they were. It is true Harry was in no way like Mr. Murray, but she liked him very much in spite of that, and her liking was in a fair way to develop into something warmer. She was happy with Harry, happy with George, and this evening as soon as one left her the other came. She had every reason to feel content. She did not even know which she liked best—Harry's anxiety to please and homage to her, or George's kind, considerate manner—a little authoritative now and then; but Edith had been used to authority all her life, and would have been lost if left to herself. This particular authority, too, was something separate and apart — something to luxuriate in and to give herself up to without reserve. She therefore complied at once with his request for a seat by her side, and he dropped into it, saying—

"What a nice place you have found out here; it seems made on purpose for two people to be happy in."

"It has been here all along," she answered. "You bought this couch yourself; Aunt Frances told me so."

"I never saw to what pleasant uses it might be put until you began to frequent it," was his reply.

She said nothing, but leaned back in her corner, her eyes fixed upon the bit of lace-work lying in her lap. Her attitude was full of consciousness, if only he had had eyes to see it; but though looking at Edith, he was thinking of Beatrice. Mrs. Ledward would have been delighted if she could have seen them; the sight of these two stricken creatures so successfully playing the game of cross purposes would have furnished her with "laughter for a month and a good jest for ever." But she was not there, and the play went on its even way unhindered. George was silent at first, but Edith did not dislike it; she was not herself inclined for conversation. Presently, however, he roused himself, and began to talk in a low tone, that Mrs. Lester might not be disturbed; and to Edith this half-whispered conversation was most delightful. This is a part of what passed between them—very ordinary words when not clothed in the glamour of a young girl's fancy and the dusk of a summer evening.

"How dark it is getting," said George. "Your white dress makes you look like a ghost in the dark, Edith. It is a very pretty dress," he went on, taking a fold between his fingers as he spoke; "what is it made of?"

"I don't know what the material is called," she answered in a low voice, startled out of her dreams.

"I don't suppose it has a name. No doubt it was imagined, and woven, and found its way into the Mayfield shops, and caught your notice all by some design, unknown to us, but which had for its object the clothing of Edith Lester in a gown which should be perfectly becoming to her."

She laughed a little nervously, but said nothing—what was there to say? His words were spoken in a half-bantering tone, and accompanied by an expression which excluded all idea of serious meaning from them; but Edith had never been quick to enter into such a mood, and as for the expression, she could not meet her companion's eyes, and so saw nothing of it.

"The way in which it is made," went on George, "is peculiar too. I have seen no dress like it. I don't believe there is one."

"Oh!" said poor Edith, almost overcome, "there is nothing peculiar about it. The town is full of dresses made in just the same way."

"Well, they don't look the same," he persisted, sticking to his point, though he could not have told why, for the subject had only the slightest degree of interest for him. "They look quite different. I suppose it is because you don't wear them, and you do wear this."

"I don't know," said Edith, vaguely. "I did not know it was so becoming."

"Oh, but it is; it suits you perfectly. It is soft, and white, and graceful, like yourself. I wonder sometimes if you know how attractive you are, Edith."

"Oh, don't!" said the girl, with a little catch of the breath. She began to feel half afraid, though she could not have said of what.

"But you are," he repeated. You go about in such a quiet, simple way, and all the time you are prettier than any girl I know. I always feel proud of having you for a companion when we go out together. I see no one with such a charming little companion."

She was silent, from excess of feeling. She sat with her face turned aside to hide her tremulous lips. She had completely forgotten Harry Fairfax now, and before any more could be said the door opened, and a maid came in with lights, and came up to the window in which they were sitting, to draw down the blind. The spell was broken—the charm gone. George rose and stretched himself, and went forward to the chimneypiece to look at the clock, after which he leaned against it, apparently lost in thought. Edith came into the middle of the room, and, seating herself near the light, took up the bit of work which she had laid aside when the daylight failed. Mrs. Lester, too, roused up from her nap and resumed her book, but they were all silent until George spoke.

"Frances," he said, addressing his sister, "have you any engagement for to-morrow afternoon?"

Mrs. Lester reflected a moment. She was always anxious to oblige her brother, even at the cost of her personal convenience—a sacrifice she made for no one else.

"I do not think there is anything," she said at last, "Edith, get my engagement-book and look."

Edith got the book, and, having consulted it, duly reported that the space assigned to the following day was blank.

"In that case," said George, "you will be at liberty to come and pay a call with me."

"Certainly," replied Mrs. Lester, with perfect amiability. "Upon whom?"

"Upon Miss Stanford, in York Gardens."

Edith almost started with surprise when she heard the name, which she had never forgotten, although she had heard it only once. She wondered what was coming, and with difficulty controlling herself so as to go on with her work as though nothing had happened, she listened eagerly to the conversation which followed.

Mrs. Lester, too, almost started with surprise. Her fears and suspicions were instantly on the alert. She was resolved to defend her post to the last. Never would she give her countenance to an intimacy with an unmarried woman. But at first she resolved to act and speak with caution,

"Miss Stanford?" she said. "Who is she? I do not know the name. I do not fancy I have ever met her."

"Probably not," returned her brother. "You go out so little, and she has only been living here about a year."

"Ah! a new comer, !" said Mrs. Lester, lifting her nose with a contemptuous sniff. "Some rich parvenue, I have no doubt. You really must not ask me to call, George; and I do hope you will be very careful how you encourage such people. Mayfield society has got very mixed of late years; I feel less and less inclined to go out."

"She is rich, certainly," said George, in the quiet way wh'ch often misled more impulsive people; "but I do not think she is a parvenue. There is nothing of that kind about her, and Stanford is a good name."

"Still, she can scarcely belong to the Stanfords of Blackmoor, or we should have heard of her before this."

"I have heard of her, as you call it, for a long time. I have known her almost ever since she came, and, as it happens, she does belong to those Stanfords; her father was Colonel Stanford, of Blackmoor."

Mrs. Lester bit her lips in anger and mortification at her own blunder. What a desirable acquaintance, if only she had not been *Miss* Stanford.

"I wonder we have not heard of her," she repeated. "It is not like a Stanford to settle down in this quiet way. Their approach is usually heralded by a flourish of trumpets."

"This member of the Stanford family is a lady," was George's reply to this, and Mrs. Lester, after a short silence, began again.

"I don't understand your wanting to call now, after you have known her, and she has been settled here a year. It looks strange. The call should have been paid at once, or not at all."

"I had reasons for not calling before, and I have reasons for calling now," he said.

"Then call," cried Mrs. Lester, goaded almost beyond endurance by this stony disregard of her feelings and opinion, "but I see no reason why my habits should be disturbed by my having to call too."

"A call from me alone, and a call from me accompanied by you, are two totally distinct things; and I wish this call to be made by both of us. You are at liberty to-morrow afternoon, so we will go then."

There was a somewhat lengthy silence, during which Mrs. Lester was digesting this intelligence. She knew when her brother had made up his mind so that she could not shake him; she knew the exact point to which opposition might be safely carried, and she was aware it had now been reached, so she was silent; and before anyone else had spoken, George rose from his leaning attitude against the mantelpiece, and left the room. A minute later they heard the study-door close behind him.

Edith had listened to all this with breathless attention and interest. She could have listened for ever, and she was sorry when Mr. Murray put an end to the discussion by leaving the room. Mrs. Lester, when the restraint of her brother's presence was withdrawn, showed one of her worst moods, and bullied Edith, until the girl was glad to go to her room rather earlier than usual.

*(To be continued.)*

